# Retraction: Reduced levels of A20 protein prompted RIPK1-dependent apoptosis and blood–brain barrier breakdown during cerebral ischemia reperfusion injury

**DOI:** 10.1371/journal.pone.0302458

**Published:** 2024-04-16

**Authors:** The PLOS ONE Editors

After this article [1] was published, concerns were raised about Figs 2A, 3A, and 5C.

Specifically:

- The WT and RIPK1-D138N panels in Fig 2A of [1] appear similar to the tMCAO and Sham panels, respectively, in Fig 2A of a previous article with no shared authors [2].
- The Sham, WT, Ripk1^D138N^ and Ripk3-/- panels in Fig 3A of [1] appear similar to the Sham, 3d, 12h and 7d panels, respectively, in Fig 1C of [2].
- The Sham, Ctrl, and A20-EKD panels in Fig 5C of [1] appear similar to the Sham, tMCAO/vehicle, and tMCAO/nec-1 panels respectively in Fig 2H of [2].
- In the S1 Raw images file, the images provided for the p-RPK1 wild type and β-actin panels in Fig 1 appear similar, when flipped horizontally, and do not match the p-RPK1 wild type data shown in the figure.
- The data in the S1 Raw images file for Fig 5 and for the Fig 4 β-actin panel do not match the results shown in the figures.

The corresponding author requested the retraction of this article [1] and stated that the underlying data was not labeled sufficiently to allow for accurate identification of the results.

In light of the above concerns, the *PLOS ONE* Editors retract this article.

MG, YL, and YW agreed with retraction. CY and XW either did not respond directly or could not be reached.
